# Disparities in the Prevalence of Urinary Diseases Among Prisoners in Taiwan: Population-Based Cross-Sectional Study

**DOI:** 10.2196/60136

**Published:** 2024-12-24

**Authors:** Yen-Chun Wang, Zhu Liduzi Jiesisibieke, Yu-Pei Yang, Bing-Long Wang, Ming-Chon Hsiung, Tao-Hsin Tung

**Affiliations:** 1 Department of Urology, Kaohsiung Medical University Hospital, Kaohsiung, Taiwan; 2 Department of Public Health, College of Medicine, National Cheng Kung University, Tainan, Taiwan; 3 Evidence-Based Medicine Center, Taizhou Hospital of Zhejiang Province Affiliated to Wenzhou Medical University, 150 Ximen Street, Gucheng Street, Linhai, Zhejiang, 317000, China, 86 13666801279; 4 Department of Hematology, Taizhou Hospital of Zhejiang Province Affiliated to Wenzhou Medical University, Linhai, Zhejiang, China; 5 School of Health Policy and Management, Chinese Academy of Medical Sciences & Peking Union Medical College, Beijing, China; 6 Department of Occupational Safety and Health, Cheng-Hsin General Hospital, Taipei, Taiwan; 7 Department of Urology, Enze Medical Center (Group), Affiliated to Hangzhou Medical College, Linhai, Zhejiang, China; 8 Key Laboratory of Evidence-based Radiology of Taizhou, Linhai, Zhejiang, China

**Keywords:** prisoners, Taiwan, health care, urinary disease, urinary tract infection, prison health

## Abstract

**Background:**

Prisoner health is a major global concern, with prisoners often facing limited access to health care and enduring chronic diseases, infectious diseases, and poor mental health due to unsafe prison environments, unhygienic living conditions, and inadequate medical resources. In Taiwan, prison health is increasingly an issue, particularly concerning urinary diseases such as urinary tract infections. Limited access to health care and unsanitary conditions exacerbate these problems. Urinary disease epidemiology varies by sex and age, yet studies in Asia are scarce, and comprehensive data on urinary diseases in Taiwanese prisons remain limited.

**Objective:**

This study aimed to investigate the prevalence of urinary diseases among Taiwanese prisoners and explore the differences in disease prevalence between men and women, as well as across different age groups.

**Methods:**

This study used data on prisoners from the National Health Insurance Research Database covering the period from January 1 to December 31, 2013. Prisoners covered by National Health Insurance who were diagnosed with urinary diseases, identified by *ICD-9-CM* (*International Classification of Diseases, Ninth Revision, Clinical Modification*) codes 580‐599 based on their medical records, and had more than one medical visit to ambulatory care or inpatient services were included. Sex- and age-stratified analyses were conducted to determine the differences in the prevalence of urinary diseases.

**Results:**

We examined 83,048 prisoners, including 2998 with urinary diseases. The overall prevalence of urinary system diseases among prisoners was 3.61% (n=2998; n=574, 6.64% in men and n=2424, 3.26% in women). The prevalence rate in men was significantly lower than that in women (prevalence ratio: 0.46, *P*<.001). In age-stratified analysis, the prevalence rate among prisoners aged >40 years was 4.5% (n=1815), compared to 2.77% (n=1183) in prisoners aged ≤40 years. Prisoners aged >40 years had a higher prevalence (prevalence ratio: 1.69, *P*<.001). Other disorders of the urethra and urinary tract (*ICD-9-CM*: 599), including urinary tract infection, urinary obstruction, and hematuria, were the most prevalent diseases of the urethra and urinary tract across age and sex groups. Women and older prisoners had a higher prevalence of most urinary tract diseases. There were no significant sex-specific differences in adjusted prevalence ratios for acute glomerulonephritis, nephrotic syndrome, kidney infections, urethritis (nonsexually transmitted), or urethral syndrome. However, based on the age-specific adjusted prevalence ratio analysis, cystitis was more prevalent among younger prisoners (prevalence ratio: 0.69, *P*=.004).

**Conclusions:**

Urinary system infections and inflammation are common in prisons. Our findings advocate for policy reforms aimed at improving health care accessibility in prisons, with a particular focus on the needs of high-risk groups such as women and older prisoners. Further research linking claims data with prisoner information is crucial to providing more comprehensive medical services and achieving health equity.

## Introduction

### Background

Inmate health is a major global concern [1]. Prisoners often lack access to health care and face injustices due to unsafe prison environments, unhygienic living conditions, and a lack of medical resources. All of these factors correlate with the high prevalence of chronic diseases, infectious diseases, and poor mental health in prisoners [2-4]. A burgeon of literature suggests a negative relationship between incarceration and health [5-7]. These health inequalities are associated with potential additional medical expenditure. Prison overcrowding correlates significantly with the spread of infectious and communicable diseases [8]. The close living quarters and limited access to health care in prisons can result in rapid disease transmission among inmates and into the community upon their release [9]. This emphasizes the importance of implementing effective public health measures in correctional facilities to minimize the impact of infectious diseases on both inmates and the general population. Moreover, incarcerated populations may be significantly exposed to extreme temperatures, as indicated by existing cases [10]. Both low and high temperatures are associated with emergency department visits for renal and urinary diseases, with heat having a stronger impact than cold. Furthermore, emergency department visits tend to decrease on days with low temperatures [11].

### Urinary Diseases

Urinary tract infections (UTIs) are among the most prevalent types of infectious diseases worldwide [12,13]. Despite their widespread occurrence, they are not currently classified as mandatory reportable conditions. The true frequency and burden of UTIs are likely underestimated due to a lack of comprehensive data on their prevalence and impact [14-16]. UTIs not only have an impact on individuals but also pose a significant public health concern. The overuse and misuse of antibiotics to treat UTIs contribute to antimicrobial resistance, making effective management more difficult. Moreover, untreated UTIs can potentially lead to more serious complications, particularly in vulnerable populations such as older adults or individuals with compromised immune systems [17]. Furthermore, a link has been discovered between early-stage chronic kidney disease (CKD) and higher health care costs over a 5-year period. Therefore, it is important to develop strategies to prevent CKD progression and reduce the associated health care expenses [18].

Understanding the differences in urinary disease epidemiology between men and women are crucial for providing sex-specific patient care. Evidence suggests that there are sex differences in the risk and outcomes of urinary disease, with mixed results depending on the cause [19,20]. Urinary disease is prevalent among individuals from disadvantaged socioeconomic backgrounds. These groups are also disproportionately affected by incarceration, and face barriers in accessing health care. Little is known about the status of urological diseases in imprisoned individuals. Some evidence suggests that the prevalence of self-reported kidney problems among incarcerated individuals varies across correctional settings; however, nuanced data characterizing urological disease trends in this population are lacking [21].

The health of prisoners in Taiwan has become a focal point, and in the past decade, the focus has shifted from solely addressing issues related to HIV, hepatitis, and other notifiable diseases to encompassing a broader range of illnesses. Data on the prevalence of diseases including oral and eye diseases are also available [22,23].

To eliminate inequalities in prisoner health, it is important to understand the specific status of each disease. This study aimed to explore the health status of Taiwanese prisoners with urinary diseases. We evaluated the prevalence of urinary diseases across different sex and age groups among prisoners in Taiwan to comprehensively review their health status, improve the identification of urinary diseases by health care professionals, and provide a basis for health policy decisions.

## Methods

### Data Source

This cross-sectional study was conducted to determine the prevalence of urinary diseases in Taiwanese prisoners, and to clarify differences in sex and age. In Taiwan, the Ministry of Health and Welfare supervises the medical care of prisoners, ensuring they receive a comprehensive initial assessment of their mental and physical health status—including medical history, current status, and past medical history—by medical professionals upon entry. All medical services accessed by prisoners are covered by the National Health Insurance program. This program in Taiwan aims to ensure the right to access health care for all individuals, regardless of their financial status, education, or race. Since March 1, 1995, the compulsory social insurance system has consisted of public and private insurance, and it covers 23,355,508 (>99%) of the Taiwanese population. The Taiwan National Health Insurance Research Database (NHIRD) records the claims data, including inpatient and outpatient records, investigations, and treatment of insured individuals [24]. The data used in this study were extracted directly from the database to prevent underestimation and bias of the actual prevalence that could occur through self-reported data obtained from incarcerated individuals.

This study used the datasets of ambulatory care expenditures by visits and datasets of inpatient expenditures by admissions from the NHIRD. We have previously investigated the prevalence of oral health and skin diseases among prison inmates in Taiwan [22,25].

### Study Design

We extracted claims data for all inmates from January 1 to December 31, 2013, including 1,122,922 person-times of outpatient data and 15,383 person-times of hospitalization data. We combined the medical records of both outpatient and inpatient visits and summarized the data for each individual. In total, this study includes 83,048 inmates. The *ICD-9-CM* (*International Classification of Diseases, Ninth Revision, Clinical Modification*) codes were used to define the diagnosis of urinary diseases [26]. Participants were identified as inmates with urinary diseases, indicated by *ICD-9* codes 580‐599 in their medical records, and who had more than one medical visit.

### Ethical Considerations

This study was conducted using data from the National Health Research Insurance Database and was approved by the Institutional Review Board Research Ethics Committee of Taizhou Hospital of Zhejiang Province (TZH-IRB: K20240836). The National Health Research Institutes encrypted the names of patients, medical care providers, and institutions, replacing the identity numbers of all insured persons with alternative unique numbers to protect their privacy. All applications for National Health Research Insurance Database access are reviewed by peer experts to ensure the rationality of their use. Researchers must comply with Taiwan’s Computer-Processed Personal Data Protection Law and related regulations when accessing the data, and they must sign an agreement declaring that they will not attempt to retrieve information about patients or medical care providers.

### Statistical Analysis

All analyses were performed using SAS (version 9.4; SAS Institute Inc). Prevalence data are expressed as numbers and percentages. The chi-square test and Fisher exact test were used to assess sex- and age-group differences in the disease percentages, and the prevalence ratio was calculated using the GENMOD procedure.

## Results

### Demographics Outcomes

**Figure 1.**
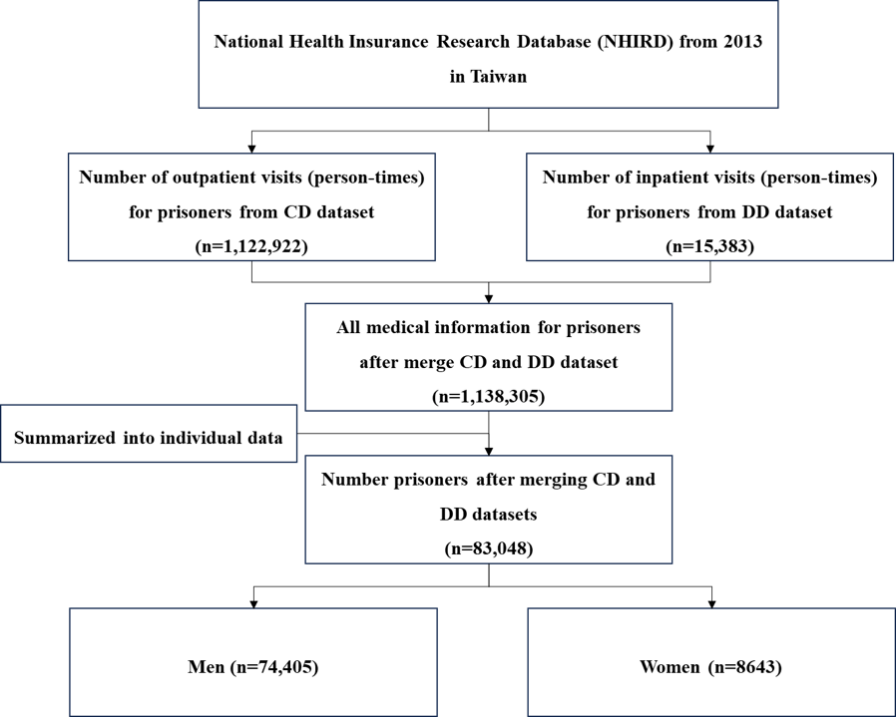
The flowchart of this study sample selection. CD: datasets of ambulatory care expenditures by visits; DD: datasets of inpatient expenditures by admissions; NHIRD: National Health Insurance Research Database.

This study included data from 83,048 prisoners (Figure 1), comprising 74,405 (89.59%) men and 8643 (10.41%) women. As shown in Table 1, 2424 male inmates were identified as having urinary system diseases, with an average age of 46.46 (SD 13.07) years and an average of 28.03 (SD 22.08) medical service visits per year. Among female inmates, 574 had urinary diseases, with an average age of 39.25 (SD 12.37) years and an average of 32.79 (SD 23.51) times per year using medical services, including outpatient and emergency services as well as hospitalization.

**Table 1.** Demographic information of the participating sample stratified by sex (Taiwan, 2013).

	Total		Diseases of urinary system	
	Women (n=8643)	Men (n=74,405)	Women (n=574)	Men (n=2424)
**Age (years)**				
Mean (SD)	38.65 (11.79)	41.50 (11.43)	39.25 (12.37)	46.46 (13.07)
Range (minimum-maximum)	2‐88	2‐103	2‐81	3‐97
**Medicine service times (a year)**				
Mean (SD)	18.17 (15.83)	13.27 (13.16)	32.79 (23.51)	28.03 (22.08)
Range (minimum-maximum)	1‐228	1‐369	2‐228	2‐369

### Prevalence of Urinary Diseases

The prevalence of urinary system disorders among the prisoners was 3.61% (n=2998; n=574, 6.64% and n=2424, 3.26% in women and men, respectively). Diseases with the highest prevalence were other disorders of the urethra and urinary tract (*ICD-9-CM*: 599), including UTI, urinary obstruction, and hematuria, which occurred in 4.65% (n=402) of women and 1.46% (n=1086) of men. The disease with the second highest prevalence differed by sex; it was cystitis (n=115, 1.33%) in women and calculus of the kidney and ureter (n=629, 0.85%) in men.

The significant sex differences in chronic renal failure (*ICD-9-CM*: 585), kidney infections (*ICD-9-CM*: 590), calculus of the kidney and ureter (*ICD-9-CM*: 592), cystitis (*ICD-9-CM*: 595), and other disorders of the urethra and urinary tract (*ICD-9-CM*: 599) are shown in (Table 2).

**Table 2.** Prevalence of diseases of the urinary system stratified by sex, based on a survey of year 2013 claims data from the Taiwan National Health Insurance program (N=83,048, Taiwan, 2013).

		Women		Men		*P* value
		n (%)	Age (years), mean (SD)	n (%)	Age (years), mean (SD)	
Total prisoners		8643 (10.41)	38.65 (11.79)	74,405 (89.59)	41.50 (11.43)	—^b^
ICD9^a^_580‐599: diseases of the urinary system		574 (6.64)	39.25 (12.37)	2424 (3.26)	46.46 (13.07)	<.001
**ICD9_580‐589: nephritis, nephrotic syndrome, and nephrosis**		32 (0.37)	47.59 (11.03)	564 (0.76)	51.44 (12.78)	<.001
	ICD9_580: acute glomerulonephritis	1 (0.01)	—^b^	5 (0.01)	39.2 (12.91)	.36
	ICD9_581: nephrotic syndrome	3 (0.03)	51.67 (12.66)	67 (0.09)	43.81 (13.29)	.09
	ICD9_582: chronic glomerulonephritis	7 (0.08)	43.57 (11.59)	45 (0.06)	51.49 (13.88)	.47
	ICD9_583: nephritis and nephropathy, not specified as acute or chronic	5 (0.06)	44.2 (15.55)	28 (0.04)	53.54 (12.29)	.13
	ICD9_584: acute renal failure	2 (0.02)	46.5 (2.12)	78 (0.1)	48.67 (14.3)	.02
	ICD9_585: chronic renal failure	17 (0.2)	49.06 (10.54)	330 (0.44)	52.79 (11.65)	.001
	ICD9_586: renal failure, unspecified	3 (0.03)	53.67 (9.02)	40 (0.05)	52.7 (11.19)	.17
	ICD9_587: renal sclerosis, unspecified	0 (0)	—^b^	1 (<0.01)	—^b^	.90
	ICD9_588: disorders resulting from impaired renal function	3 (0.03)	43.67 (10.07)	45 (0.06)	55.89 (11.47)	.14
	ICD9_589: small kidney of unknown cause	0 (0)	—^b^	0 (0)	—^b^	—^b^
**ICD9_590‐599: other diseases of urinary system**		550 (6.36)	38.8 (12.22)	1922 (2.58)	45.25 (12.94)	<.001
	ICD9_590: infections of kidney	45 (0.52)	37.33 (13.23)	58 (0.08)	43.88 (10.39)	<.001
	ICD9_591: hydronephrosis	12 (0.14)	40.17 (8.45)	168 (0.23)	44.93 (10.9)	.10
	ICD9_592: calculus of kidney and ureter	39 (0.45)	43.85 (10.41)	629 (0.85)	45.65 (10.68)	<.001
	ICD9_593: other disorders of kidney and ureter	10 (0.12)	41.4 (12.32)	56 (0.08)	50.54 (14.38)	.21
	ICD9_594: calculus of lower urinary tract	2 (0.02)	53.5 (4.95)	39 (0.05)	49.33 (13.66)	.12
	ICD9_595: cystitis	115 (1.33)	37.76 (12.23)	143 (0.19)	38.9 (11.4)	<.001
	ICD9_596: other disorders of bladder	16 (0.19)	36.75 (12.8)	171 (0.23)	45.46 (14.41)	.41
	ICD9_597: urethritis, not sexually transmitted, and urethral syndrome	10 (0.12)	41.6 (12.37)	63 (0.08)	39.78 (12.6)	.36
	ICD9_598: urethral stricture	0 (0)	—^b^	16 (0.02)	51.44 (14.91)	—^b^
	ICD9_599: other disorders of urethra and urinary tract	402 (4.65)	38.92 (12.42)	1086 (1.46)	45.37 (13.61)	<.001

a *ICD9*: *International Classification of Diseases, Ninth Revision*.

b Not applicable.

Age-specific prevalence rates are shown in Table 3. There were significant differences in the prevalences of most disease classifications except for acute glomerulonephritis (*ICD-9-CM*: 580), nephrotic syndrome (*ICD-9-CM*: 581), renal sclerosis (*ICD-9-CM*: 587), hydronephrosis (*ICD-9-CM*: 591), and urethritis (not sexually transmitted), and urethral syndrome (*ICD-9-CM*: 597). In prisoners aged ≤40 years, three of the most common diseases were other disorders of the urethra and urinary tract (n=665, 1.56%), calculus of the kidney and ureter (n=223, 0.52%), and cystitis (n=165, 0.39%). Among men, the top three diseases were other disorders of the urethra and urinary tract (n=823, 2.04%), calculus of the kidney and ureter (n=445, 1.10%), and chronic renal failure (n=287, 0.71%).

**Table 3.** Prevalence of diseases of the urinary system stratified by age group, based on a survey of year 2013 claims data from the Taiwan National Health Insurance program (N=83,048, Taiwan, 2013).

		Aged ≤40 years		Aged >40 years		*P* value
		n (%)	Age (years), mean (SD)	n (%)	Age (years), mean (SD)	
Total prisoners		42,684 (51.4)	32.30 (6.16)	40,364 (48.6)	50.62 (7.71)	—^b^
ICD9^a^_580‐599: diseases of the urinary system		1183 (2.77)	32.34 (6.19)	1815 (4.5)	53.38 (9.49)	<.001
**ICD9_580‐589: nephritis, nephrotic syndrome, and nephrosis**		135 (0.32)	34.39 (4.95)	461 (1.14)	56.16 (9.72)	<.001
	ICD9_580: acute glomerulonephritis	4 (0.01)	34.00 (6.48)	2 (<0.01)	58.50 (2.12)	.25
	ICD9_581: nephrotic syndrome	35 (0.08)	32.91 (4.81)	35 (0.09)	55.37 (8.65)	.82
	ICD9_582: chronic glomerulonephritis	15 (0.04)	35.00 (4.72)	37 (0.09)	56.68 (10.97)	.001
	ICD9_583: nephritis and nephropathy, not specified as acute or chronic	7 (0.02)	33.43 (4.72)	26 (0.06)	57.15 (9.33)	.001
	ICD9_584: acute renal failure	24 (0.06)	33.13 (5.14)	56 (0.14)	55.25 (11.21)	.001
	ICD9_585: chronic renal failure	60 (0.14)	35.23 (4.83)	287 (0.71)	56.24 (9.04)	<.001
	ICD9_586: renal failure, unspecified	6 (0.01)	36.33 (3.67)	37 (0.09)	55.43 (9.28)	<.001
	ICD9_587: renal sclerosis, unspecified	0 (0)	—^b^	1 (<0.01)	—^b^	.49
	ICD9_588: disorders resulting from impaired renal function	7 (0.02)	37.57 (2.43)	41 (0.1)	58.12 (9.82)	<.001
	ICD9_589: small kidney of unknown cause	0 (0)	—^b^	0 (0)	—^b^	—^b^
**ICD9_590‐599: other diseases of urinary system**		1065 (2.5)	32.12 (6.27)	1407 (3.49)	52.67 (9.38)	<.001
	ICD9_590: infections of kidney	50 (0.12)	31.16 (7.94)	53 (0.13)	50.32 (6.80)	.56
	ICD9_591: hydronephrosis	71 (0.17)	34.69 (4.04)	109 (0.27)	51.07 (8.72)	.001
	ICD9_592: calculus of kidney and ureter	223 (0.52)	34.69 (4.61)	445 (1.1)	50.99 (8.44)	<.001
	ICD9_593: other disorders of kidney and ureter	19 (0.04)	33.63 (5.56)	47 (0.12)	55.43 (11.85)	<.001
	ICD9_594: calculus of lower urinary tract	12 (0.03)	35.92 (1.88)	29 (0.07)	55.17 (11.89)	<.001
	ICD9_595: cystitis	165 (0.39)	31.12 (5.68)	93 (0.23)	51.28 (8.17)	<.001
	ICD9_596: other disorders of bladder	80 (0.19)	31.15 (4.77)	107 (0.27)	54.85 (10.37)	.02
	ICD9_597: urethritis, not sexually transmitted, and urethral syndrome	44 (0.1)	31.82 (5.37)	29 (0.07)	52.48 (9.53)	.13
	ICD9_598: urethral stricture	3 (0.01)	31.33 (3.79)	13 (0.03)	56.08 (12.30)	.009
	ICD9_599: other disorders of urethra and urinary tract	665 (1.56)	31.77 (6.59)	823 (2.04)	53.20 (9.70)	<.001

a *ICD9*: *International Classification of Diseases, Ninth Revision*.

b Not applicable.

Age- and sex-specific adjusted prevalence ratios were calculated and are presented in Table 4. Higher prevalence rates adjusted by age were observed in men for acute and chronic renal failure, calculus of the kidney and ureter, and other disorders of the urethra and urinary tract. In contrast, women had higher prevalences of kidney infection and cystitis compared to men. The sex-adjusted prevalence ratios were significantly >1 for most diseases, except for acute glomerulonephritis, nephrotic syndrome, kidney infections, urethritis (nonsexually transmitted), and urethral syndrome. Additionally, the prevalence of cystitis was lower in inmates aged >40 years compared to that in younger individuals.

**Table 4.** Differences in medical use among prisoners. Reference group: sex: women; age: >40 years (N=83,048).

		n (%)	PR^_sex_^^a^	*P* ^_sex_^	PR^_age_^	*P* ^_age_^
ICD9^b^_580‐599: diseases of the urinary system		2998 (3.61)	0.46	<.01	1.69	<.001
**ICD9_580‐589: nephritis, nephrotic syndrome, and nephrosis**		596 (0.72)	1.8	<.01	3.54	<.001
	ICD9_580: acute glomerulonephritis	6 (0.01)	0.62	.66	0.54	.48
	ICD9_581: nephrotic syndrome	70 (0.08)	2.59	.11	1.03	.90
	ICD9_582: chronic glomerulonephritis	52 (0.06)	0.67	.33	2.66	.002
	ICD9_583: nephritis and nephropathy, not specified as acute or chronic	33 (0.04)	0.57	.24	4.04	.001
	ICD9_584: acute renal failure	80 (0.1)	4.13	.048	2.38	<.001
	ICD9_585: chronic renal failure	347 (0.42)	1.93	<.01	4.96	<.001
	ICD9_586: renal failure, unspecified	43 (0.05)	1.31	.66	6.46	<.001
	ICD9_587: renal sclerosis, unspecified	1 (<0.01)	—^c^	—^c^	—^c^	—^c^
	ICD9_588: disorders resulting from impaired renal function	48 (0.06)	1.47	.52	6.11	<.001
	ICD9_589: small kidney of unknown cause	0 (0)	—^c^	—^c^	—^c^	—^c^
**ICD9_590‐599: other diseases of urinary system**		2472 (2.98)	0.39	<.01	1.47	<.001
	ICD9_590: infections of kidney	103 (0.12)	0.15	<.01	1.30	.19
	ICD9_591: hydronephrosis	180 (0.22)	1.55	.15	1.69	.002
	ICD9_592: calculus of kidney and ureter	668 (0.8)	1.73	<.01	2.07	<.001
ICD9_593: other disorders of kidney and ureter		66 (0.08)	0.59	.12	2.69	<.001
	ICD9_594: calculus of lower urinary tract	41 (0.05)	2.06	.32	2.50	.008
	ICD9_595: cystitis	258 (0.31)	0.15	<.01	0.69	.004
	ICD9_596: other disorders of bladder	187 (0.23)	1.20	.49	1.41	.02
	ICD9_597: urethritis, not sexually transmitted, and urethral syndrome	73 (0.09)	0.76	.42	0.71	.15
	ICD9_598: urethral stricture	16 (0.02)	—^c^	—^c^	4.38	.02
	ICD9_599: other disorders of urethra and urinary tract	1488 (1.79)	0.30	<.01	1.41	<.001

a PR: prevalence ratio.

b *ICD9*: *International Classification of Diseases, Ninth Revision*.

c Not applicable.

## Discussion

### Principal Findings

The provision of adequate medical care for incarcerated individuals is not only a matter of basic human rights but also a public health concern. Ensuring access to necessary health care services and providing a healthy environment can help prevent the spread of infectious diseases within correctional facilities and the broader community, as well as reduce the consumption of medical resources. In addition, addressing prisoners’ health care needs can contribute to their successful reintegration into society. Therefore, we conducted an analysis to determine the prevalence of urinary diseases in Taiwanese prisons.

The results of this study showed that most diseases with high prevalence were related to infection and inflammation of the urinary tract. Approximately 3.6% of the inmates had urinary diseases, and their prevalence differed according to sex and age. To our knowledge, this is the first study to explore urinary system diseases in Taiwanese inmates.

The annual statistics showed that the prevalence of nephritis, nephrotic syndrome, and nephrosis in the Taiwanese general population was 2.32%, with 2.61% in men and 2.03% in women [27]. The community-based screening program also found a higher CKD prevalence in the general population than in prisons [28]. This disparity might reflect that the use of medical services for urinary diseases in prisons differs from the actual prevalence, indicating that many prisoners do not receive appropriate medical care. Consequently, this may overestimate the health status and quality of life of prisoners with urinary diseases, indicating that their conditions could be worse than previously thought.

Consistent with previous research, we found that infections and inflammation of the urinary system are more prevalent in women (women: n=574, 6.64%, men: n=2424, 3.26%), and that the prevalence increases with age (>40 years: n=1815, 4.5% and ≤40 years: n=1183, 2.77%). Biological sex (women or men) widely influences various immune responses, including immune responses to mucosal surface diseases. Sex hormones, sex chromosomes, sexual dimorphism, and sexual differences all affect an organism’s response to urological diseases such as bladder infections or cancer [19,29]. Women are more likely to experience urinary system diseases, particularly infections. We infer the following reasons based on previous literatures. Anatomical differences, such as the shorter female urethra and its proximity to the anus, make women more prone to UTIs [30]. Additionally, pregnancy and menopause can increase the risk of urinary incontinence, pelvic organ prolapse, and other urinary system disorders [31,32]. Furthermore, the female reproductive system, including the uterus and ovaries, can affect the urinary system and contribute to the development of diseases [33]. These conditions can significantly impact a woman’s quality of life, leading to urinary symptoms, sexual dysfunction, and more serious complications. They also place a significant economic burden on health care systems due to costs associated with diagnosis, treatment, and management [34-39]. In contrast, there is a higher risk of severe kidney disease in men than in women [40,41]. One key factor is the higher prevalence of conditions such as diabetes and high blood pressure, which are leading causes of kidney disease [42]. Men are more likely than women to develop these conditions, putting them at greater risk for kidney damage. Additionally, lifestyle factors such as smoking, poor diet, and lack of exercise can also contribute to the higher prevalence of end-stage kidney disease in men [43]. Another key factor is the genetic and hormonal differences between men and women. Research has shown that certain genetic factors may predispose men to kidney disease, while hormonal differences, such as the effects of testosterone, may also influence kidney function [44]. Understanding these biological differences is essential for developing targeted interventions to reduce the risk of different urinary system diseases [45].

As individuals age, the risk of developing urinary system diseases is more likely to increase due to a decline in urinary system function and the presence of other age-related health conditions [46,47]. Older adults face a higher risk of UTIs, stones, benign prostatic hyperplasia, and other urinary system disorders, which may lead to discomfort, urination problems, pain, and complications requiring medical treatment [48,49]. The impact of aging on urinary system diseases can result in higher health care costs and decreased quality of life, ultimately affecting overall health and well-being [50,51].

According to our findings, the prevalence of urinary diseases is mainly attributable to infection. Incarcerated individuals are at a higher risk of developing UTIs due to various factors such as limited access to health care, poor hygiene practices, and overcrowded living conditions [52]. UTIs are common and can affect the bladder, kidneys, ureters, and urethra. Antimicrobial resistance is a growing concern in the treatment of UTIs. The overuse and misuse of antibiotics, along with limited access to medical resources, have contributed to the development of resistant bacteria, making it more difficult to treat these infections [53]. UTIs pose significant challenges for individuals and health care systems regarding personal health and financial resources in the era of antimicrobial resistance [54,55].

Measures to reduce the medical burden of UTIs among prisoners should include prevention, diagnosis, and treatment. Providing a healthy environment, ensuring adequate water intake, and dietary interventions such as the provision of cranberries can help reduce the incidence of UTIs [56-60]. Personal hygiene is also essential in minimizing the risk of UTIs. Regular medical screenings and access to medical care are crucial for identifying and treating UTIs in their early stages, and a well-equipped and adequately staffed health care system within correctional facilities is required to treat them [61]. This is a multifaceted endeavor that requires a combination of education, access to resources, and comprehensive health care services. By adopting a comprehensive approach to UTI prevention—including general hygiene practices and targeted medical interventions—prisoners’ overall health and well-being can be significantly improved.

### Strengths

We used claims data from the NHIRD instead of prisoner questionnaires to prevent underestimation of the prevalence of urinary diseases and to avoid selection bias. The results were based on repeated diagnoses using harmonized *ICD* codes, minimizing measurement bias and improving the comparability of our findings.

### Limitations

Our study had some limitations. First, this was a Taiwanese population-based cross-sectional study, so our findings may not be generalizable to other regions and races. Additionally, the results were based solely on claims data from the NHIRD in 2013, which could not identify any causality between the incidence of urinary diseases and imprisonment. Second, we could not obtain certain information related to urinary diseases such as socioeconomic status and biochemical factors. Furthermore, we were unable to distinguish between the duration and facility of imprisonment and criminal type to clarify the relationship between urinary diseases and imprisonment. Third, although we used data from the NHIRD instead of self-reports, the prevalence of urinary diseases is still likely to have been underestimated, as discrimination by prison officials may have prevented inmates from receiving medical services. Furthermore, the precision of diagnoses based on *ICD* codes can differ greatly from diseases, and *ICD* codes may not always accurately reflect the true clinical status, leading to potential misclassification.

### Conclusions

Our study revealed sex- and age-related discrepancies in the prevalence of urinary diseases. We found that the prevalence of urinary system diseases is related to infection and inflammation. These findings emphasize the importance of diagnosis and treatment. Thus, considering these findings, comprehensive medical services that implement specialized intervention strategies or policies are needed in prisons to enhance health equality among Taiwanese prisoners and reduce their overall medical burden. Future studies should include more factors influencing health care use and the incidence of urinary diseases among prisoners to investigate ways to improve their overall quality of life and health in the prison.

## Abbreviations

CKD: chronic kidney disease
*ICD-9-CM*: *International Classification of Diseases, Ninth Revision, Clinical Modification*
NHIRD: National Health Insurance Research Database
UTI: urinary tract infection
